# Retraction Note: Differences in cognitive functions between cytomegalovirus-infected and cytomegalovirus-free university students: a case control study

**DOI:** 10.1038/s41598-022-09409-0

**Published:** 2022-03-29

**Authors:** Veronika Chvátalová, Blanka Šebánková, Hana Hrbáčková, Petr Tureček, Jaroslav Flegr

**Affiliations:** 1 Laboratory of Evolutionary Biology, Department of Philosophy and History of Sciences, Faculty of Science, Charles University, Vinicna 7, 128 00, Prague 2, Czech Republic; 2 National Reference Laboratory for Herpesviruses, National Institute of Public Health, Prague, Srobarova 48, 100 42, Prague 10, Czech Republic

Retraction of: *Scientific Reports* 10.1038/s41598-018-23637-3, published online 28 March 2018

The authors are retracting this article.

After the publication of the article, J. Gottfried and H. Cigler brought to our attention that the results of the permutation tests for contaminated data in the explorative part of the study were incorrect. We checked the program used in the present study (and recommended by us to be used in future studies) to find out that it has illogically coded infected (0) and uninfected (1) individuals. Due to this error, our explanation of why the CMV-infected subjects have on average higher intelligence than the CMV-free subjects (due to contamination of the later subset with false-negative individuals with old infections and therefore very low levels of both anti-CMV antibodies and intelligence) was wrong. Therefore, the results of our permutation tests falsify, rather than support, the suggested model.

Based on these facts, we retract the article and suggest that future studies search for another explanation of the observed paradox of higher intelligence of CMV-infected subjects. We also recommend, whenever possible, to substitute the old version of our program Treept which was not user-friendly with other programs, e.g. with our new and more complex program for computing permutation tests for contaminated data^1^.

Veronika Chvátalová, Blanka Šebánková, Petr Tureček, and Jaroslav Flegr agree with this retraction and its wording. Hana Hrbáčková did not respond to correspondence from the Editors about this retraction.
